# COVID-19–related health literacy, general health literacy, and mental health problems: evidence from a population-based study in Japan

**DOI:** 10.1265/ehpm.26-00003

**Published:** 2026-06-30

**Authors:** Keiko Murakami, Hideki Hashimoto

**Affiliations:** 1 Department of Health and Social Behavior, School of Public Health, The University of Tokyo, 7-3-1 Hongo, Bunkyo-ku, Tokyo 113-0033, Japan; 2 Tohoku Medical Megabank Organization, Tohoku University, 2-1 Seiryo-machi, Aoba-ku, Sendai, Miyagi 980-8573, Japan

**Keywords:** COVID-19, Health literacy, Japan, Mental health conditions, Pandemic

## Abstract

**Background:**

The coronavirus disease 2019 (COVID-19) pandemic highlighted poor health literacy (HL) as an underestimated public health problem. This study aimed to examine whether COVID-19–related HL and general HL were differentially associated with mental health problems.

**Methods:**

A questionnaire survey was conducted among residents of Japanese metropolitan areas during the resurgence phase of the pandemic. Data from 1,482 participants were analyzed. COVID-19–related HL was assessed by participants’ perceived difficulty in understanding COVID-19–related information and recommendations. General HL was measured using the Communicative and Critical Health Literacy scale. Mental health outcomes included psychological distress, mood and anxiety disorders, and COVID-19–related anxiety. Multiple logistic regression analyses were conducted to estimate odds ratios (ORs) and 95% confidence intervals (CIs) per one–standard deviation increase in each HL measure, adjusting for age, sex, education, income, work status, self-rated health, and the other HL measure.

**Results:**

The correlation coefficient between the two HL measures was 0.195. Higher COVID-19–related HL was associated with decreased risks of mental health problems; the ORs were 0.70 (95% CI, 0.62–0.78) for psychological distress, 0.76 (95% CI, 0.66–0.88) for mood and anxiety disorders, and 0.66 (95% CI, 0.53–0.81) for COVID-19–related anxiety. General HL was not associated with mental health problems; the corresponding ORs were 0.95 (95% CI, 0.85–1.06), 0.90 (95% CI, 0.79–1.03), and 1.11 (95% CI, 0.90–1.36), respectively.

**Conclusions:**

Higher COVID-19–related HL, but not general HL, was associated with decreased risks of mental health problems.

**Supplementary information:**

The online version contains supplementary material available at https://doi.org/10.1265/ehpm.26-00003.

## Background

The unprecedented worldwide crisis caused by the rapid spread of coronavirus disease 2019 (COVID-19), along with the restrictive public health measures enforced to contain and slow its transmission, has affected all aspects of people’s daily lives and severely threatened the physical and mental wellbeing of communities globally. In fact, the global prevalence and burden of major depressive and anxiety disorders increased substantially during the first year of the COVID-19 pandemic [1]. This increase can be explained by multiple factors, including fear of infection, social isolation, low education, financial hardship, unemployment, and insufficient knowledge of COVID-19 [2, 3].

COVID-19 is the first pandemic in history in which technology and social media have been used on a massive scale to keep people safe, informed, productive, and connected [4]. At the same time, the technology relied upon to stay connected and informed has enabled and amplified an infodemic—an overabundance of information—that continues to undermine the global response and jeopardize measures to control the pandemic [4, 5]. Health literacy (HL) acts as a means to understand and apply information about protection against the coronavirus [6], and the COVID-19 infodemic has highlighted that poor HL within a population is an underestimated public health problem worldwide [7]. There is some evidence that levels of HL are associated with mental health problems during the COVID-19 pandemic [8–19]. However, these studies have focused mainly on general HL. This type of HL may be inadequate for examining the role of HL more broadly because HL is context-dependent. In fact, we previously reported that COVID-19–related HL and general HL were differently associated with protective behaviors [20]. To the best of our knowledge, no studies have simultaneously examined the associations of general HL and COVID-19–related HL with mental health problems.

Given the abovementioned circumstances, we aimed to examine whether COVID-19–related HL and general HL were differently associated with mental health problems, hypothesizing that higher levels of COVID-19–related HL, rather than general HL, would be associated with decreased risks of mental health problems.

## Methods

### Study design and participants

Data were derived from the Japanese Study of Stratification, Health, Income, and Neighborhood (J-SHINE), which has been described in detail elsewhere [21]. The baseline survey of J-SHINE was conducted in four municipalities in and around the greater Tokyo metropolitan area between October 2010 and February 2011 and involved residents aged 25–50 years who were probabilistically selected from the residential registry of each municipality. The second and third surveys were conducted in 2012 and 2017, respectively [22]. A supplementary survey was conducted between December 2020 and January 2021 to assess the socioeconomic and health impacts of the COVID-19 pandemic [20, 23]. The present study was a cross-sectional analysis based on data from this supplementary J-SHINE survey, although information on general HL obtained at the first-wave survey was also used. Survey invitations were sent to 3,062 residents whose contact information had been obtained in the 2017 survey. Of these, 1,638 residents responded to the online or paper-based questionnaire. We analyzed data from 1,482 participants with no missing values for any variables used in the analyses.

### COVID-19–related HL and general HL

COVID-19–related HL was measured based on the questionnaire from the survey tool and guidance edited by the World Health Organization Regional Office for Europe [24]. Participants were asked how easy or difficult it was to find necessary COVID-19–related information, understand information on what to do if they thought they got infected with COVID-19, judge whether COVID-19–related information in the media was reliable, understand government restrictions and recommendations regarding COVID-19, follow recommendations on how to protect themselves from COVID-19, understand recommendations on when to stay at home from work or school, and understand recommendations on when to engage in social activities. The conceptual basis of COVID-19–related HL lies in assessing individuals’ perspectives on access to, understanding of, and use of information and knowledge [24]. Each item was rated from 1 (very difficult) to 5 (very easy). The scores of the seven items were summed and divided by the number of items to produce a total score (theoretical range: 1–5), with higher scores indicating greater HL [20]. The Cronbach’s alpha value of this scale was 0.85.

General HL was measured using the Communicative and Critical Health Literacy (CCHL) scale [25]. Communicative and critical literacy extends beyond basic skills in reading and writing to function effectively in everyday situations [26]. Participants were asked whether they could perform the following tasks in the context of health issues: collect information from various sources, extract the information they wanted, understand and communicate the obtained information, consider the credibility of the information, and make decisions based on the information. Each item was rated from 1 (strongly disagree) to 5 (strongly agree). The scores of the five items were summed and divided by the number of items to produce a total score (theoretical range: 1–5), with higher scores indicating greater HL [20]. The Cronbach’s alpha value of this scale was 0.83.

### Mental health problems

The K6 scale consists of six items asking respondents how often they felt nervous, hopeless, restless or fidgety, so depressed that nothing could cheer them up, that everything was an effort, and worthless during the past month [27, 28]. Response options were all of the time, most of the time, some of the time, a little of the time, and none of the time, coded from 4 to 0. Total scores therefore ranged from 0 to 24. A cut-off score of 5 was used to identify psychological distress [29], and a cut-off score of 9 was used to identify mood and anxiety disorders [27]. The optimal cut-off point has been estimated as 4/5 for K6 in Japan [29].

COVID-19–related anxiety was measured using four items from the Coronavirus Anxiety Scale: dizziness, sleep disturbance, appetite loss, and abdominal distress when thinking about or being exposed to information on COVID-19 during the past month [30]. Response options were all of the time, most of the time, some of the time, little of the time, and none of the time. As most respondents answered “little of the time” or “none of the time” for each item, participants who answered “some of the time,” “most of the time,” or “all of the time” to at least one item were classified as having COVID-19–related anxiety.

### Covariates

Covariates included age, sex, educational attainment (high school or lower, college, university or higher), annual household income (≤3.99, 4.00–7.49, 7.50–9.99, ≥10.00 million Japanese yen, unknown/refusal), work status (working, not working), and self-rated health. Participants were asked to describe their health over the past month, and responses were dichotomized into good health (excellent, very good, good) and poor health (fair, poor).

### Statistical analysis

Participant characteristics were compared between low and high categories of HL (dichotomized at the median) using chi-squared tests. Multiple logistic regression analyses were conducted to examine associations between COVID-19–related HL, general HL, and mental health outcomes. Odds ratios (ORs) and 95% confidence intervals (CIs) for a one–standard deviation (SD) increase in each HL measure were calculated after adjustment for covariates (model 2a for COVID-19–related HL and model 2b for general HL). COVID-19–related HL and general HL were then entered simultaneously into the same model (model 3). Sensitivity analyses were conducted by categorizing HL into four groups after dichotomizing each HL measure at the median: low level in both HLs, high level in general HL only, high level in COVID-19–related HL only, and high level in both HLs [20].

All analyses were conducted using Stata version 16.0 (StataCorp LP, College Station, TX, USA). A two-tailed *P* value of <0.05 was considered statistically significant for all analyses.

## Results

Table 1 shows the participants’ characteristics. The prevalence of psychological distress, mood and anxiety disorders, and COVID-19–related anxiety was 43.5%, 20.2%, and 6.8%, respectively. Participants with high COVID-19–related HL were more likely to be men, have higher educational attainment, and report higher household income than those with low COVID-19–related HL. Supplementary Table 1 shows the participants’ characteristics by general HL level (Additional file 1). The mean scores were 3.19 (SD, 0.62) for COVID-19–related HL and 3.65 (SD, 0.61) for general HL. The correlation coefficient between COVID-19–related HL and general HL was 0.195.

**Table 1:** Characteristics of participants: the Japanese Study of Stratification, Health, Income, and Neighborhood (J-SHINE)

	**Total** **(N = 1482)**	**COVID-19–related HL**		

		**Low** **(n = 756)**	**High** **(n = 726)**	***P*-value^a^**
	**n (%)**	**n (%)**	**n (%)**	
Age				0.64
35–44 years	470 (31.7)	240 (31.8)	230 (31.7)	
45–54 years	671 (45.3)	335 (44.3)	336 (46.3)	
55–61 years	341 (23.0)	181 (23.9)	160 (22.0)	
Women	817 (55.1)	441 (58.3)	376 (51.8)	0.011
Educational attainment				0.005
High school or lower	284 (19.2)	164 (21.7)	120 (16.5)	
College	511 (34.5)	270 (35.7)	241 (33.2)	
University or higher	687 (46.3)	322 (42.6)	365 (50.3)	
Annual household income				<0.001
≤3.99 million Japanese yen	349 (23.6)	199 (26.3)	150 (20.7)	
4.00–7.49 million Japanese yen	379 (25.6)	184 (24.3)	195 (26.9)	
7.50–9.99 million Japanese yen	291 (19.6)	140 (18.5)	151 (20.8)	
≥10.00 million Japanese yen	289 (19.5)	126 (16.7)	163 (22.4)	
Unknown/refusal	174 (11.7)	107 (14.2)	67 (9.2)	
Working	1311 (88.5)	668 (88.4)	643 (88.6)	0.90
Poor self-rated health	145 (9.8)	74 (9.8)	71 (9.8)	0.995
Psychological distress	644 (43.5)	375 (49.6)	269 (37.1)	<0.001
Mood and anxiety disorders	300 (20.2)	179 (23.7)	121 (16.7)	0.001
COVID-19–related anxiety	100 (6.8)	69 (9.1)	31 (4.3)	<0.001

HL, health literacy.

^a^Obtained using the chi-squared test, comparing low and high groups of COVID-19–related HL.

Table 2 presents the associations between COVID-19–related HL, general HL, and psychological distress. Higher COVID-19–related HL scores were associated with a decreased risk of psychological distress (model 2a); the OR was 0.69 (95% CI, 0.62–0.78). This association remained after further adjustment for general HL (model 3); the OR was 0.70 (95% CI, 0.62–0.78). Higher general HL scores were associated with a decreased risk of psychological distress (model 2b); the OR was 0.89 (95% CI, 0.80–0.99). This association disappeared after further adjustments for COVID-19–related HL (model 3); the OR was 0.95 (95% CI, 0.85–1.06). Lower household income and poor self-rated health were associated with an increased risk of psychological distress. High level in COVID-19–related HL only and high level in both HLs, not high level in general HL only, were associated with decreased risks of psychological distress (Supplementary Table 2; Additional file 2).

**Table 2:** Associations between COVID-19–related HL, general HL, and psychological distress

	**Psychological distress**	**Model 1**	**Model 2a**	**Model 2b**	**Model 3**
	**%**	**OR (95% CI)**	**OR (95% CI)**	**OR (95% CI)**	**OR (95% CI)**
COVID-19–related HL (1–SD increase)		0.69 (0.62–0.77)	0.69 (0.62–0.78)		0.70 (0.62–0.78)
General HL (1–SD increase)		0.86 (0.78–0.96)		0.89 (0.80–0.99)	0.95 (0.85–1.06)
Age					
35–44 years	46.0	1.00	1.00	1.00	1.00
45–54 years	42.9	0.88 (0.70–1.12)	0.95 (0.73–1.22)	0.92 (0.72–1.19)	0.96 (0.74–1.23)
55–61 years	41.1	0.82 (0.62–1.09)	0.81 (0.60–1.09)	0.85 (0.63–1.14)	0.82 (0.60–1.10)
Sex					
Men	41.8	1.00	1.00	1.00	1.00
Women	44.8	1.13 (0.92–1.39)	1.03 (0.81–1.30)	1.07 (0.85–1.35)	1.03 (0.81–1.30)
Educational attainment					
High school or lower	47.2	1.00	1.00	1.00	1.00
College	43.3	0.85 (0.64–1.14)	0.84 (0.61–1.15)	0.82 (0.61–1.12)	0.84 (0.61–1.15)
University or higher	42.1	0.81 (0.62–1.07)	0.96 (0.71–1.30)	0.93 (0.69–1.26)	0.97 (0.71–1.31)
Annual household income					
≤3.99 million Japanese yen	52.4	1.00	1.00	1.00	1.00
4.00–7.49 million Japanese yen	42.5	0.67 (0.50–0.90)	0.74 (0.55–1.01)	0.71 (0.53–0.96)	0.75 (0.55–1.02)
7.50–9.99 million Japanese yen	36.1	0.51 (0.37–0.70)	0.55 (0.39–0.77)	0.53 (0.38–0.74)	0.55 (0.39–0.77)
≥10.00 million Japanese yen	35.0	0.49 (0.35–0.67)	0.57 (0.41–0.80)	0.55 (0.40–0.78)	0.58 (0.41–0.82)
Unknown/refusal	54.0	1.07 (0.74–1.53)	1.09 (0.75–1.60)	1.13 (0.78–1.65)	1.10 (0.75–1.62)
Work status					
Working	43.0	1.00	1.00	1.00	1.00
Not working	46.8	1.16 (0.85–1.60)	1.06 (0.74–1.50)	0.98 (0.69–1.38)	1.05 (0.74–1.49)
Self-rated health					
Good	39.9	1.00	1.00	1.00	1.00
Poor	75.9	4.73 (3.18–7.02)	4.84 (3.22–7.27)	4.67 (3.12–6.97)	4.83 (3.22–7.27)

CI, confidence interval; HL, health literacy; OR, odds ratio; SD, standard deviation.

Model 1: crude.

Model 2a (for COVID-19–related HL) and 2b (for general HL): adjusted for age, sex, educational attainment, annual household income, work status, and self-rated health.

Model 3: model 2 + adjusted for general HL/COVID-19–related HL.

Table 3 presents the associations between COVID-19–related HL, general HL, and mood and anxiety disorders. Higher COVID-19–related HL scores were associated with a decreased risk of mood and anxiety disorders (model 2a); the OR was 0.75 (95% CI, 0.65–0.86). This association remained after further adjustments for general HL (model 3); the OR was 0.76 (95% CI, 0.66–0.88). Higher general HL scores were associated with a decreased risk of mood and anxiety disorders (model 2b); the OR was 0.86 (95% CI, 0.76–0.98). This association disappeared after further adjustments for COVID-19–related HL (model 3); the OR was 0.90 (95% CI, 0.79–1.03). Younger age, lower household income, and poor self-rated health were associated with an increased risk of mood and anxiety disorders. High level in COVID-19–related HL only and high level in both HLs, not high level in general HL only, were associated with decreased risks of mood and anxiety disorders (Supplementary Table 2; Additional file 2).

**Table 3:** Associations between COVID-19–related HL, general HL, and mood and anxiety disorders

	**Mood and anxiety** **disorders**	**Model 1**	**Model 2a**	**Model 2b**	**Model 3**
	**%**	**OR (95% CI)**	**OR (95% CI)**	**OR (95% CI)**	**OR (95% CI)**
COVID-19–related HL (1–SD increase)		0.74 (0.65–0.84)	0.75 (0.65–0.86)		0.76 (0.66–0.88)
General HL (1–SD increase)		0.81 (0.72–0.92)		0.86 (0.76–0.98)	0.90 (0.79–1.03)
Age					
35–44 years	25.1	1.00	1.00	1.00	1.00
45–54 years	18.8	0.69 (0.52–0.92)	0.72 (0.53–0.98)	0.71 (0.52–0.97)	0.73 (0.53–0.99)
55–61 years	16.4	0.59 (0.41–0.84)	0.56 (0.38–0.82)	0.59 (0.40–0.86)	0.57 (0.39–0.84)
Sex					
Men	19.4	1.00	1.00	1.00	1.00
Women	20.9	1.10 (0.85–1.42)	0.99 (0.74–1.32)	1.01 (0.76–1.34)	0.98 (0.74–1.31)
Educational attainment					
High school or lower	22.5	1.00	1.00	1.00	1.00
College	20.2	0.87 (0.61–1.23)	0.84 (0.57–1.23)	0.83 (0.57–1.22)	0.84 (0.58–1.23)
University or higher	19.4	0.83 (0.59–1.16)	0.94 (0.65–1.37)	0.94 (0.65–1.36)	0.96 (0.66–1.40)
Annual household income					
≤3.99 million Japanese yen	28.7	1.00	1.00	1.00	1.00
4.00–7.49 million Japanese yen	19.0	0.58 (0.41–0.83)	0.64 (0.45–0.93)	0.62 (0.43–0.90)	0.65 (0.45–0.94)
7.50–9.99 million Japanese yen	15.1	0.44 (0.30–0.66)	0.48 (0.32–0.73)	0.47 (0.31–0.72)	0.49 (0.32–0.74)
≥10.00 million Japanese yen	12.5	0.35 (0.23–0.54)	0.43 (0.28–0.67)	0.43 (0.28–0.67)	0.45 (0.29–0.70)
Unknown/refusal	27.6	0.95 (0.63–1.42)	1.03 (0.67–1.59)	1.06 (0.69–1.63)	1.05 (0.68–1.62)
Work status					
Working	19.9	1.00	1.00	1.00	1.00
Not working	22.8	1.19 (0.81–1.74)	1.05 (0.68–1.60)	0.98 (0.64–1.50)	1.02 (0.67–1.57)
Self-rated health					
Good	16.8	1.00	1.00	1.00	1.00
Poor	52.4	5.47 (3.83–7.81)	5.53 (3.81–8.01)	5.42 (3.75–7.83)	5.52 (3.81–8.01)

CI, confidence interval; HL, health literacy; OR, odds ratio; SD, standard deviation.

Model 1: crude.

Model 2a (for COVID-19–related HL) and 2b (for general HL): adjusted for age, sex, educational attainment, annual household income, work status, and self-rated health.

Model 3: model 2 + adjusted for general HL/COVID-19–related HL.

Table 4 presents the associations between COVID-19–related HL, general HL, and COVID-19–related anxiety. Higher COVID-19–related HL scores were associated with a decreased risk of COVID-19–related anxiety (model 2a); the OR was 0.67 (95% CI, 0.54–0.82). This association remained after further adjustments for general HL (model 3); the OR was 0.66 (95% CI, 0.53–0.81). General HL scores were not associated with COVID-19–related anxiety (model 2b, model 3); the ORs were 1.04 (95% CI, 0.85–1.27) and 1.11 (95% CI, 0.90–1.36), respectively. Lower household income and poor self-rated health were associated with an increased risk of COVID-19–related anxiety. High level in COVID-19–related HL only and high level in both HLs, not high level in general HL only, were associated with decreased risks of COVID-19–related anxiety (Supplementary Table 2; Additional file 2).

**Table 4:** Associations between COVID-19–related HL, general HL, and COVID-19–related anxiety

	**COVID-19–related** **anxiety**	**Model 1**	**Model 2a**	**Model 2b**	**Model 3**
	**%**	**OR (95% CI)**	**OR (95% CI)**	**OR (95% CI)**	**OR (95% CI)**
COVID-19–related HL (1–SD increase)		0.64 (0.52–0.78)	0.67 (0.54–0.82)		0.66 (0.53–0.81)
General HL (1–SD increase)		0.94 (0.77–1.15)		1.04 (0.85–1.27)	1.11 (0.90–1.36)
Age					
35–44 years	7.9	1.00	1.00	1.00	1.00
45–54 years	5.7	0.70 (0.44–1.12)	0.73 (0.45–1.18)	0.68 (0.42–1.11)	0.72 (0.44–1.17)
55–61 years	7.3	0.93 (0.55–1.57)	0.89 (0.52–1.54)	0.89 (0.52–1.54)	0.88 (0.51–1.52)
Sex					
Men	5.1	1.00	1.00	1.00	1.00
Women	8.1	1.63 (1.06–2.50)	1.34 (0.85–2.12)	1.37 (0.87–2.17)	1.35 (0.85–2.14)
Educational attainment					
High school or lower	9.2	1.00	1.00	1.00	1.00
College	7.8	0.84 (0.50–1.41)	0.84 (0.49–1.44)	0.81 (0.48–1.39)	0.84 (0.49–1.43)
University or higher	5.0	0.52 (0.30–0.88)	0.65 (0.37–1.14)	0.61 (0.35–1.06)	0.63 (0.36–1.11)
Annual household income					
≤3.99 million Japanese yen	9.7	1.00	1.00	1.00	1.00
4.00–7.49 million Japanese yen	6.6	0.65 (0.38–1.12)	0.79 (0.45–1.36)	0.71 (0.41–1.23)	0.77 (0.45–1.34)
7.50–9.99 million Japanese yen	5.2	0.50 (0.27–0.94)	0.62 (0.33–1.17)	0.57 (0.30–1.08)	0.60 (0.32–1.15)
≥10.00 million Japanese yen	2.8	0.26 (0.12–0.58)	0.37 (0.16–0.82)	0.33 (0.15–0.73)	0.35 (0.16–0.79)
Unknown/refusal	10.3	1.07 (0.59–1.95)	1.05 (0.57–1.96)	1.02 (0.55–1.88)	1.03 (0.55–1.92)
Work status					
Working	6.3	1.00	1.00	1.00	1.00
Not working	10.5	1.76 (1.03–3.02)	1.40 (0.79–2.48)	1.34 (0.76–2.36)	1.43 (0.81–2.54)
Self-rated health					
Good	6.1	1.00	1.00	1.00	1.00
Poor	12.4	2.17 (1.26–3.73)	1.96 (1.12–3.43)	1.99 (1.15–3.46)	1.98 (1.13–3.46)

CI, confidence interval; HL, health literacy; OR, odds ratio; SD, standard deviation.

Model 1: crude.

Model 2a (for COVID-19–related HL) and 2b (for general HL): adjusted for age, sex, educational attainment, annual household income, work status, and self-rated health.

Model 3: model 2 + adjusted for general HL/COVID-19–related HL.

## Discussion

The present study examined associations between COVID-19–related HL, general HL, and mental health conditions in Japan during the COVID-19 pandemic. Higher COVID-19–related HL was associated with decreased risks of psychological distress, mood and anxiety disorders, and COVID-19–related anxiety. Higher general HL was associated with decreased risks of psychological distress and mood and anxiety disorders; however, these associations disappeared after adjustment for COVID-19–related HL. General HL was not associated with COVID-19–related anxiety.

Associations between higher COVID-19–related HL and decreased risks of mental health problems remained even after adjustment for general HL. Only few studies have examined associations between COVID-19–related HL and mental health problems [11]. One online survey conducted in Japan in September 2020 showed that higher COVID-19–related HL assessed using a COVID-19–adapted CCHL scale was associated with decreased risks of anxiety (K6 ≥10) and depressive symptoms (Edinburgh Postnatal Depression Scale score ≥13) among pregnant women in Japan, although general HL was not adjusted for [11]. In the J-SHINE survey, we previously categorized HL into four groups and showed that high level in COVID-19–related HL only was associated with greater adherence to COVID-19 protective behaviors [20]. This suggests that individuals with adequate general HL may still face HL challenges in the pandemic. Another study in Japan demonstrated that COVID-19–related HL reduced belief in COVID-19- and vaccination-related misinformation, although it had no effect on belief in COVID-19–related conspiracy theories [31]. One review emphasized the need to develop HL instruments specific to COVID-19 [32].

Several mechanisms may explain why higher COVID-19–related HL was associated with decreased risks of mental health problems. The situation during a newly emerging pandemic is challenged by substantial scientific uncertainty [33]. This J-SHINE supplementary survey was conducted during the third wave of the pandemic, when the number of infections exceeded peaks observed during earlier waves [34], suggesting that uncertainty regarding many aspects of COVID-19 persisted. In such contexts, judging and applying COVID-19–related information is particularly challenging and may exacerbate mental health problems especially among individuals with lower COVID-19–related HL. One study during the early phase of the pandemic in China showed that resilience mediated the association between infection disease-specific HL and anxiety [18], although the present study did not examine this mediating effect because of the lack of resilience item in the J-SHINE survey. The clarification of the mechanism underlying the association between COVID-19–related HL and mental health problems should be needed.

Associations between higher general HL and decreased risks of mental health problems disappeared after adjustment for COVID-19–related HL. This finding is inconsistent with accumulated evidence that higher levels of general HL were associated with decreased risks of mental health problems [8–10, 12, 14–17, 19]. One possible explanation is whether analyses adjust for COVID-19–related HL; to the best of our knowledge, the present study is the first to examine the association between general HL and mental health while accounting for COVID-19–related HL. Indeed, the present study found the associations of general HL with psychological distress and mood and anxiety disorders before adjusting for COVID-19–related HL. Another possible explanation for this inconsistency is that general HL in the J-SHINE survey was measured about ten years before the COVID-19 pandemic. HL can change over time [35, 36], and one study showed that general HL declined significantly from immediately before the COVID-19 pandemic to one year later [37]. Nevertheless, we previously showed that higher general HL in the first wave of J-SHINE was associated with healthy balanced diet, adequate sleep, and regular exercise during the COVID-19 pandemic [20]. By contrast, one study in Japan found greater COVID-19–related anxiety among individuals with higher general HL assessed by the CCHL and interpreted this unexpected finding that such individuals may be more cautious and more informed about risks and hazards [13]. Differences in survey timing may partly explain these inconsistencies; that study was conducted during the early phase of the pandemic (between late April and May 2020), characterized by greater uncertainty and complexity [13], while the J-SHINE survey was conducted during the resurgence of the pandemic (between December 2020 and January 2021). A systematic review rated the overall evidence linking HL and mental health outcomes as low [38], indicating that these associations may be more complex than expected.

The correlation coefficient between COVID-19–related HL and general HL was relatively weak. One possible explanation is a time discrepancy, as general HL was measured about ten years before the COVID-19 pandemic. Another possible explanation is that these two measures reflect different aspects of HL. The context-specific characteristics of HL suggest that even people with generally adequate HL can face HL challenges in uncertain situations.

These findings have implications for governments and health authorities struggling with mental health problems during the pandemic. Information oversaturation makes it difficult to distinguish accurate from inaccurate information, facilitating the spread of misconceptions and wrong beliefs, many times under the cover of almost scientific speech. In addition to this, simply knowing about the risks (i.e. ‘functional’ health literacy) is insufficient when scientific knowledge is still limited. Our findings suggest that general HL may be inadequate for assessing responses to pandemics caused by new viruses or infodemics. There is an urgent need to develop validated and standardized instruments for rapid assessment of infectious disease–specific HL during the pandemic alongside general HL. Because HL is mediated by cultural and situational demands placed on individuals, organizations, and society, it is not solely an individual responsibility [39]. One study in Japan showed that HL assessed using the CCHL scale improved following a community-based educational program [40]. Although evidence is lacking for similar interventions targeting infectious disease–specific HL, educational efforts during the early phase of the pandemic may help individuals develop skills to access, understand, appraise, and apply health information.

Several limitations of this study should be noted. First, the causal direction of the observed associations cannot be inferred because of the cross-sectional design; mental health problems can impair the ability to process COVID-19–related information. Second, the findings should only be generalized with caution, because the survey was conducted in and around the Tokyo metropolitan area, which experienced the highest infection burden in Japan, and findings may not apply to other countries with different cultural and social contexts. Third, the response rate was not high; the overall response rate was 37.2% compared with the first survey. While the participants of the J-SHINE survey in the first wave were comparable with the vital statistics of the target population in terms of age, sex, and educational attainment [21], the fourth-wave participants were more likely to be female and more highly educated than non-participants [23]. However, sex and educational attainment were not associated with mental health outcomes in the present study. Fourth, the reliability and validity of the questionnaire for COVID-19–related HL have not been confirmed, although this questionnaire was adapted from the European Health Literacy Survey Questionnaire [41]. There is no gold-standard instrument which evaluates COVID-19–related HL. The development and validation of such a tool is desirable, as an increase in the levels of COVID-19–related HL is necessary beyond governmental regulations and policy [32]. Fifth, the HL measures were not contemporaneous: COVID-19–related HL was measured during the COVID-19 pandemic, whereas general HL was measured approximately ten years earlier. Finally, the psychometric validity of the COVID-19–related anxiety scale has not been confirmed.

## Conclusions

The present study showed that higher COVID-19–related HL was associated with decreased risks of psychological distress, mood and anxiety disorders, and COVID-19–related anxiety, even after adjustment for general HL, whereas general HL was not associated with these outcomes. Although the causal direction of the observed associations cannot be determined, these findings suggest that incorporating infectious disease–specific HL into public health strategies may facilitate a better understanding of mental health during the pandemic, with consequent preparation for possible future pandemics and public health crises.

## Abbreviations

CCHL: Communicative and Critical Health Literacy
COVID-19: coronavirus disease 2019
HL: health literacy
J-SHINE: Japanese Study of Stratification, Health, Income, and Neighborhood
OR: odds ratio
95% CI: 95% confidence interval

## Supplementary information

**Additional file 1:** **Supplementary Table 1.** Characteristics of participants by general HL level.

**Additional file 2:** **Supplementary Table 2.** Associations of combinations of COVID-19–related HL and general HL with mental health problems.

## Declarations

### Availability of data and material

J-SHINE data will be made available after approval for data use by the J-SHINE Data Management Committee.
