# Supplementary material for: COVID-19–related health literacy, general health literacy, and mental health problems: evidence from a population-based study in Japan
Source: Environ Health Prev Med. 2026 Jun 30;31:40. doi: 10.1265/ehpm.26-00003 (PMC13366167; doi:10.1265/ehpm.26-00003)
Supplement: Supplementary file 1 — Additional file 1: Supplementary Table 1. Characteristics of participants by general HL level. [file ehpm-31-040-s001.docx]

**Additional file 1**

| **Supplementary Table 1. Characteristics of participants by general HL level** | | | | | |
| --- | --- | --- | --- | --- | --- |
|  | general HL | | | | |
|  | Low (n=920) | | High (n=562) | | *P*-value^a^ |
|  | n (%) | | n (%) | |  |
| Age |  |  |  |  | 0.008 |
| 35–44 years | 317 | (34.5) | 153 | (27.2) |  |
| 45–54 years | 408 | (44.3) | 263 | (46.8) |  |
| 55–61 years | 195 | (21.2) | 146 | (26.0) |  |
| Women | 524 | (57.0) | 293 | (52.1) | 0.070 |
| Educational attainment |  |  |  |  | 0.005 |
| High school or lower | 189 | (20.6) | 95 | (16.9) |  |
| College | 335 | (36.4) | 176 | (31.3) |  |
| University or higher | 396 | (43.0) | 291 | (51.8) |  |
| Annual household income |  |  |  |  | <0.001 |
| ≤3.99 million Japanese yen | 239 | (26.0) | 110 | (19.6) |  |
| 4.00–7.49 million Japanese yen | 241 | (26.2) | 138 | (24.5) |  |
| 7.50–9.99 million Japanese yen | 182 | (19.8) | 109 | (19.4) |  |
| ≥10.00 million Japanese yen | 144 | (15.6) | 145 | (25.8) |  |
| Unknown/refusal | 114 | (12.4) | 60 | (10.7) |  |
| Working | 806 | (87.6) | 505 | (89.9) | 0.19 |
| Poor self-rated health | 92 | (10.0) | 53 | (9.4) | 0.72 |
| Psychological distress | 423 | (46.0) | 221 | (39.3) | 0.012 |
| Mood and anxiety disorders | 201 | (21.9) | 99 | (17.6) | 0.049 |
| COVID-19–related anxiety | 67 | (7.3) | 33 | (5.9) | 0.29 |
| HL, health literacy. | | | | | |
| ^a^Obtained using the chi-squared test, comparing low and high groups of general HL. | | | | | |
