# Supplementary material for: COVID-19–related health literacy, general health literacy, and mental health problems: evidence from a population-based study in Japan
Source: Environ Health Prev Med. 2026 Jun 30;31:40. doi: 10.1265/ehpm.26-00003 (PMC13366167; doi:10.1265/ehpm.26-00003)
Supplement: Supplementary file 2 — Additional file 2: Supplementary Table 2. Associations of combinations of COVID-19–related HL and general HL with mental health problems. [file ehpm-31-040-s002.docx]

**Additional file 2**

| **Supplementary Table 2. Associations of combinations of COVID-19–related HL and general HL with mental health problems** | | | | | | | | | |
| --- | --- | --- | --- | --- | --- | --- | --- | --- | --- |
|  | Psychological distress | | | Mood and anxiety disorders | | | COVID-19–related anxiety | | |
|  | OR (95% CI) | | | OR (95% CI) | | | OR (95% CI) | | |
| COVID-19–related HL and general HL |  |  |  |  |  |  |  |  |  |
| Low level in both HLs | 1.00 |  |  | 1.00 |  |  | 1.00 |  |  |
| High level in general HL only | 0.74 | (0.54–1.03) | | 0.79 | (0.53–1.18) | | 1.13 | (0.66–1.96) | |
| High level in COVID-19–related HL only | 0.56 | (0.43–0.74) | | 0.61 | (0.43–0.87) | | 0.55 | (0.31–0.95) | |
| High level in both HLs | 0.56 | (0.42–0.75) | | 0.63 | (0.43–0.92) | | 0.47 | (0.24–0.90) | |
| CI, confidence interval; HL, health literacy; OR, odds ratio. | | | | | | | | | |
| Adjusted for age, sex, educational attainment, annual household income, work status, and self-rated health. | | | | | | | | | |
